# Supplementary material for: Mechanism of outer membrane destabilization by global reduction of protein content
Source: Nat Commun. 2023 Sep 15;14:5715. doi: 10.1038/s41467-023-40396-6 (PMC10504340; doi:10.1038/s41467-023-40396-6)
Supplement: Supplementary file 1 — Supplementary Info [file 41467_2023_40396_MOESM1_ESM.pdf]

Supplemental Figures

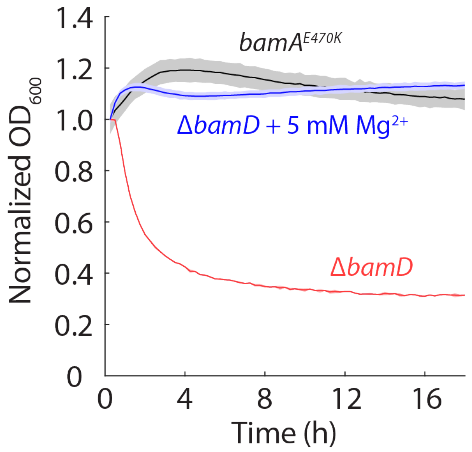

Figure S1: Mg^{2+} addition prevents lysis of Δ*bamD* cells in spent LB. OD was measured after log-phase cells in LB were transferred to spent LB. OD measurements were normalized to the value upon transfer at *t*=0. Curves are mean values and shading represents 1 standard deviation. *n*=2 biological replicates.

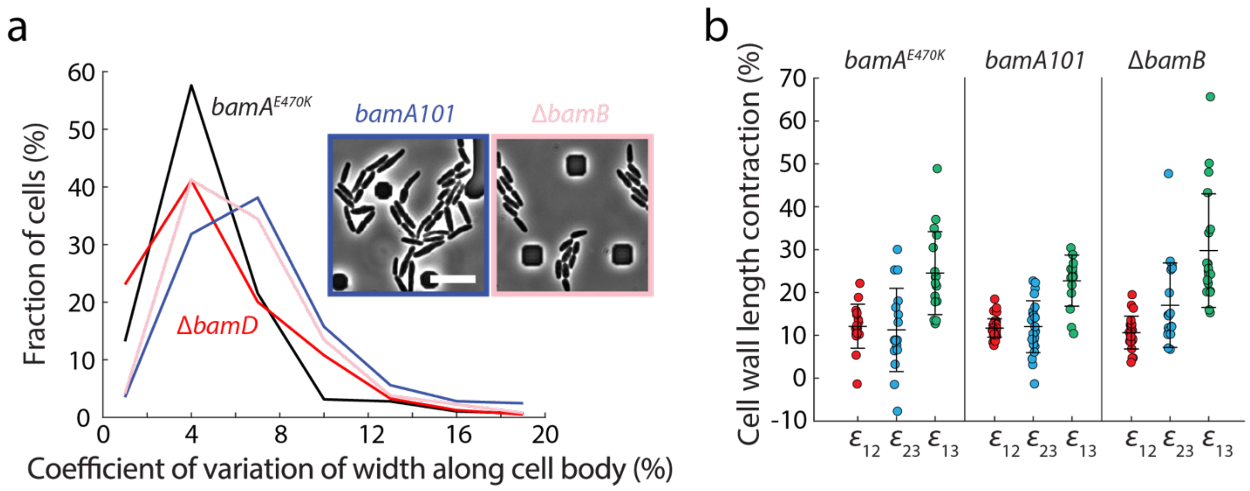

Figure S2: Introduction of the *bamA101* allele or deletion of *bamB* causes cell shape defects without compromising OM stiffness.

a) Similar to Δ*bamD* cells, *bamA101* and Δ*bamB* cells exhibit more width defects than *bamA*^{E470K}, as indicated by the increased fraction of cells with larger coefficient of variation of cell width. Cells were grown in a microfluidic device; square regions are pillars in the device. Scale bar: 10 µm. *n*>260 cells for each strain.

b) *bamA101* and Δ*bamB* cells have similar OM stiffness to *bamA*^{E470K} cells, as indicated by the similar length contractions (ε_{12}, ε_{23}, and ε_{13}) upon plasmolysis, lysis, and in total, respectively. Data are presented as mean values ±1 standard deviation. Sample sizes: *n*=17 cells for ε_{12}, ε_{23}, and ε_{13} for *bamA*^{E470K}; *n*=39, 15, and 24 for ε_{12}, ε_{23}, and ε_{13}, respectively, for *bamA101*; *n*=23, 19, and 19 for ε_{12}, ε_{23}, and ε_{13}, respectively, for Δ*bamB*.

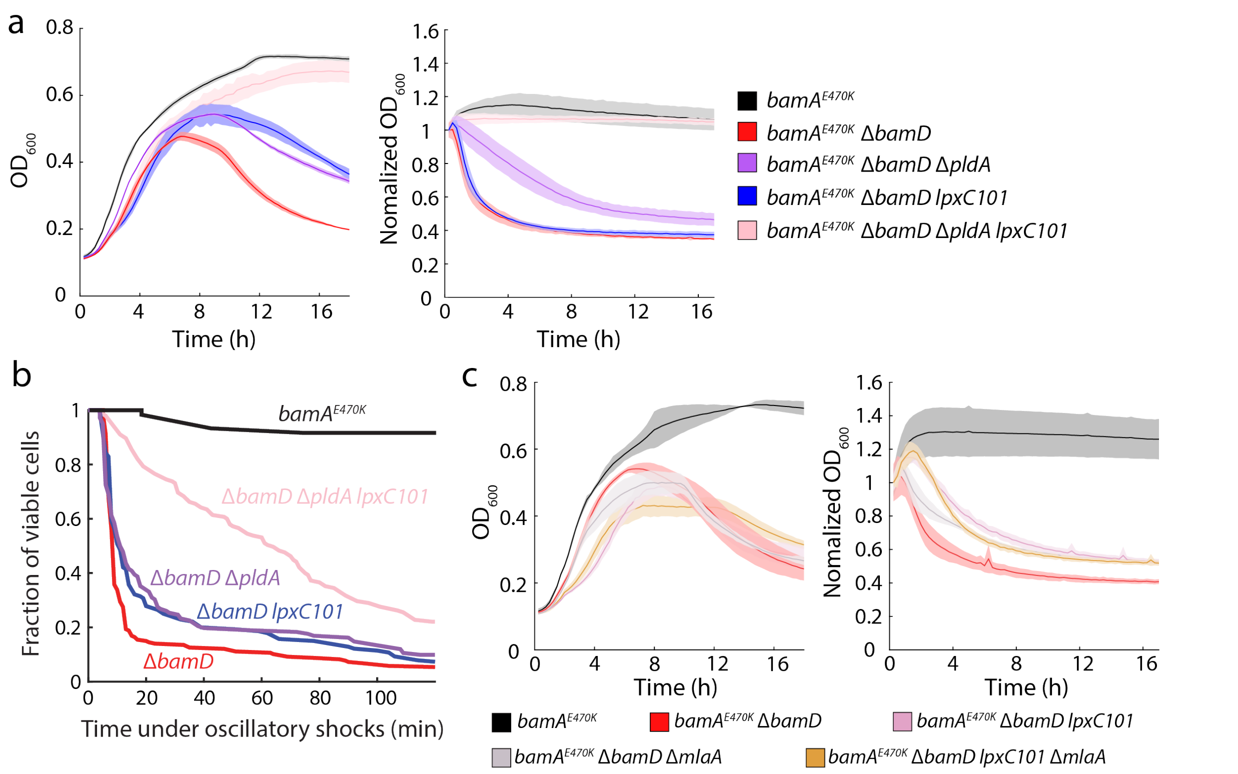

Figure S3: Introduction of the *lpxC101* allele to Δ*bamD* cells improves cell growth and rescues lysis in spent media similar to deletion of *mlaA*.

a) Lysis of Δ*bamD* cells in stationary phase (left) and spent LB (right) was rescued by introduction of the *lpxC101* allele and deletion of *pldA*, while *lpxC101* alone did not suppress lysis in spent LB. *n*=3 biological replicates. Curves are mean values, and shading represents 1 standard deviation.

b) Cell lysis due to oscillatory hyperosmotic shocks (cycles of 1 min in LB and 1 min in LB+400 mM sorbitol) (Methods) was partially rescued by the *lpxC101* allele and deletion of *pldA*, while *lpxC101* alone did not suppress lysis. *n*=60 for *bamA*^{E470K}; *n*>100 cells for all other strains.

c) Lysis of Δ*bamD* cells in stationary phase (left) or spent LB (right) was not suppressed by the *lpxC101* allele, deletion of *mlaA*, or both. *n*=3 biological replicates and shading represents 1 standard deviation.

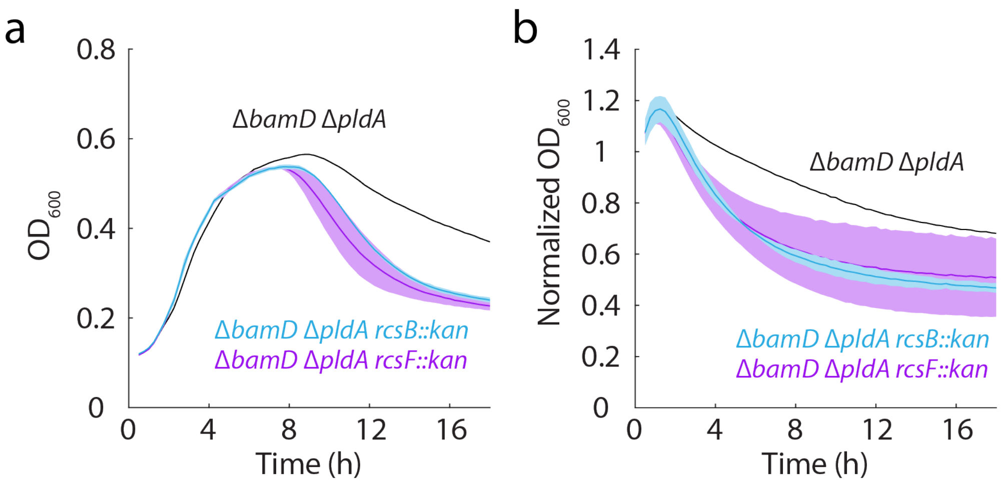

Figure S4: Disruption of the Rcs pathway does not reduce cell lysis due to *bamD* deletion.

a,b) Disruption of the Rcs pathway by deleting either *rcsB* or *rcsF* from Δ*bamD* Δ*pldA* cells did not reduce cell lysis in stationary phase cultures (a) or in log-phase cultures transferred to spent LB (b). Shading represents 1 standard deviation. *n*=2 biological replicates for Δ*bamD* Δ*pldA rcsB::kan* and Δ*bamD* Δ*pldA rcsF::kan*; *n* = 1 biological replicate for Δ*bamD* Δ*pldA*.

Supplementary Table

Strain	Genotype	Source/reference
MC4100	F-*araD139* (*argF-lac*)*U169 rpsL150 relA1 flb5301 deoC1 ptsF25 thi*	Ref. [41]
JCM158	MC4100 *ara*^{R}	Ref. [42]
HC687	MC4100 *ara*$^{+}$ *mlaA** Δ*yfdI*	Ref. [15]
IMB1047	JCM158 *bamA*^{E470K}	This study
IMB1056	JCM158 *bamA*^{E470K} Δ*bamD*	This study
IMB1132	JCM158 *bamA*^{E470K} Δ*bamD* Δ*pldA*	This study
IMB1089	JCM158 *bamA*^{E470K} Δ*bamD lpxC101 leuB::kan*	This study
IMB1090	JCM158 *bamA*^{E470K} Δ*bamD lpxC101 leuB::tn10*	This study
IMB1139	JCM158 *bamA*^{E470K} Δ*bamD* Δ*pldA lpxC101 leuB::tn10*	This study
IMB1199	JCM158 *bamA*^{E470K} Δ*bamD* Δ*mlaA lpxC101 leuB::tn10*	This study
IMB1235	JCM158 *bamA*^{E470K} Δ*bamD* Δ*mlaA*	This study
IMB1231	JCM158 *bamA*^{E470K} Δ*bamD* Δ*pldA* Δ*mlaA*	This study
IMB1233	JCM158 *bamA*^{E470K} Δ*bamD* Δ*pldA* Δ*mlaC*	This study
IMB1130	JCM158 *bamA*^{E470K} *lptD-3xFLAG*	This study
IMB1131	JCM158 *bamA*^{E470K} Δ*bamD lptD-3xFLAG*	This study
IMB1241	JCM158 *bamA*^{E470K} Δ*bamD lptD-3xFLAG lpxC101 leuB::kan*	This study
IMB1242	JCM158 *bamA*^{E470K} Δ*bamD lptD-3xFLAG mlaA::kan*	This study

IMB1243	JCM158 *bamA*^{E470K} Δ*bamD lptD-3xFLAG pldA::kan*	This study
IMB1247	JCM158 *bamA*^{E470K} Δ*bamD lptD-3xFLAG* Δ*pldA lpxC101 leuB::kan*	This study
IMB1248	JCM158 *bamA*^{E470K} Δ*bamD lptD-3xFLAG* Δ*pldA mlaA::kan*	This study
IMB1143	JCM158 *bamA*^{E470K} *attHK::Plac-mCherry pZS21kan::GFP*	This study
IMB1144	JCM158 *bamA*^{E470K} Δ*bamD attHK::Plac-mCherry pZS21kan::GFP*	This study
IMB1210	JCM158 *bamA*^{E470K} Δ*bamD lpxC101 leuB::tn10 attHK::Plac-mCherry pZS21kan::GFP*	This study
IMB1211	JCM158 *bamA*^{E470K} Δ*bamD* Δ*pldA attHK::Plac-mCherry pZS21kan::GFP*	This study
IMB1212	JCM158 *bamA*^{E470K} Δ*bamD lpxC101 leuB::tn10 attHK::Plac-mCherry pZS21kan::GFP*	This study
IMB1239	JCM158 *bamA*^{E470K} Δ*bamD* Δ*mlaA attHK::Plac-mCherry pZS21kan::GFP*	This study
IMB1240	JCM158 *bamA*^{E470K} Δ*bamD* Δ*pldA* Δ*mlaA attHK::Plac-mCherry pZS21kan::GFP*	This study
IMB1202	JCM158 *bamA*^{E470K} Δ*bamD* Δ*pldA rcsB::kan*	This study
IMB1203	JCM158 *bamA*^{E470K} Δ*bamD* Δ*pldA rcsF::kan*	This study
IMB479	JCM158 *bamA101*	This study
BH92	JCM158 *bamB::kan* (Blattner allele)	This study
**Plasmids**		
pCP20	Temperature sensitive vector expressing yeast Flp recombinase	Ref. [43]
pZS21	Low-copy vector with kanamycin resistance	Ref. [44]
pZS21::GFP	Vector expressing cytoplasmic GFP	Ref. [45]

Supplementary Table 1: ***E. coli*** **strains and plasmids used in this study.**

	Δ*bamD*, CV: (0,4)	*bamA*^{E470K}, CV: (4,8)	Δ*bamD*, CV: (4,8)	*bamA*^{E470K}, CV: (8,12)	Δ*bamD*, CV: (8,12)	*bamA*^{E470K}, CV: (12,16)	Δ*bamD*, CV: (12,16)	Δ*bamD*, CV: (16,30)
bamA^{E470K}, CV: (0,4)	8.2×10^{-8}	0.040		0.31		0.18		
Δ*bamD*, CV: (0,4)			0.52		0.061		0.0068	1.2×10^{-7}
bamA^{E470K}, CV: (4,8)			7.4×10^{-7}	0.94		0.41		
Δ*bamD*, CV: (4,8)					0.0036		0.027	1.8×10^{-7}
bamA^{E470K}, CV: (8,12)					0.0025	0.56		
Δ*bamD*, CV: (8,12)							0.21	1.9×10^{-5}
bamA^{E470K}, CV: (12,16)							0.64	
Δ*bamD*, CV: (12,16)								0.0057

Supplementary Table 2: Pairwise *p*-values from two-sample Kolmogorov-Smirnov tests in Figure 2b.
