# Supplementary material for: Mechanism of outer membrane destabilization by global reduction of protein content
Source: Nat Commun. 2023 Sep 15;14:5715. doi: 10.1038/s41467-023-40396-6 (PMC10504340; doi:10.1038/s41467-023-40396-6)
Supplement: Supplementary file 3 — Description of Additional Supplementary Files [file 41467_2023_40396_MOESM3_ESM.pdf]

Description of Additional Supplementary Files:

Supplementary Movie 1: Time-lapse movies for log-phase *bamA*^{E470K} cells growing on agarose pads with LB. Frame interval is 1 min.

Supplementary Movie 2: Time-lapse movies for log-phase Δ*bamD* cells growing on agarose pads with LB. Frame interval is 1 min.

Supplemental Movie 3: Time-lapse movie of log-phase Δ*bamD* cells lysing under oscillatory hyperosmotic osmotic shocks in a microfluidic flow cell. Oscillatory shocks start at *t*=0. Frame interval is 1 min.
