# Supplementary material for: Mechanism of outer membrane destabilization by global reduction of protein content
Source: Nat Commun. 2023 Sep 15;14:5715. doi: 10.1038/s41467-023-40396-6 (PMC10504340; doi:10.1038/s41467-023-40396-6)
Supplement: Supplementary file 7 — Reporting Summary [file 41467_2023_40396_MOESM7_ESM.pdf]

nature portfolio

Corresponding author(s): Kerwyn Casey Huang, Thomas J. Silhavy

Last updated by author(s): Jul 18, 2023

Reporting Summary

Nature Portfolio wishes to improve the reproducibility of the work that we publish. This form provides structure for consistency and transparency in reporting. For further information on Nature Portfolio policies, see our Editorial Policies and the Editorial Policy Checklist.

Statistics

For all statistical analyses, confirm that the following items are present in the figure legend, table legend, main text, or Methods section.

n/a	Confirmed	
☐	☒	The exact sample size (*n*) for each experimental group/condition, given as a discrete number and unit of measurement
☐	☒	A statement on whether measurements were taken from distinct samples or whether the same sample was measured repeatedly
☐	☒	The statistical test(s) used AND whether they are one- or two-sided<br>*Only common tests should be described solely by name; describe more complex techniques in the Methods section.*
☒	☐	A description of all covariates tested
☒	☐	A description of any assumptions or corrections, such as tests of normality and adjustment for multiple comparisons
☐	☒	A full description of the statistical parameters including central tendency (e.g. means) or other basic estimates (e.g. regression coefficient) AND variation (e.g. standard deviation) or associated estimates of uncertainty (e.g. confidence intervals)
☐	☒	For null hypothesis testing, the test statistic (e.g. *F*, *t*, *r*) with confidence intervals, effect sizes, degrees of freedom and *P* value noted<br>*Give P values as exact values whenever suitable.*
☒	☐	For Bayesian analysis, information on the choice of priors and Markov chain Monte Carlo settings
☒	☐	For hierarchical and complex designs, identification of the appropriate level for tests and full reporting of outcomes
☐	☒	Estimates of effect sizes (e.g. Cohen's *d*, Pearson's *r*), indicating how they were calculated

Our web collection on statistics for biologists contains articles on many of the points above.

Software and code

Policy information about availability of computer code

Data collection	Imaging data were collected using the open source software µManager v. 1.4. Microfluidics data were collected using the software CellASIC ONIX2.
Data analysis	An open source MATLAB software, Morphometrics (v. 1.2, https://simtk.org/projects/morphometrics), an image segmentation tool, DeepCell, (the initial version is described in the publication https://journals.plos.org/ploscompbiol/article?id=10.1371/journal.pcbi.1005177), and custom MATLAB code were used for image analysis.

For manuscripts utilizing custom algorithms or software that are central to the research but not yet described in published literature, software must be made available to editors and reviewers. We strongly encourage code deposition in a community repository (e.g. GitHub). See the Nature Portfolio guidelines for submitting code & software for further information.

Data

Policy information about availability of data

All manuscripts must include a data availability statement. This statement should provide the following information, where applicable:

- Accession codes, unique identifiers, or web links for publicly available datasets
- A description of any restrictions on data availability
- For clinical datasets or third party data, please ensure that the statement adheres to our policy

All data used for generating figures in this study are available at the Stanford Digital Repository https://purl.stanford.edu/gp172gf6221.

Research involving human participants, their data, or biological material

Policy information about studies with human participants or human data. See also policy information about sex, gender (identity/presentation), and sexual orientation and race, ethnicity and racism.

Reporting on sex and gender	N/A
Reporting on race, ethnicity, or other socially relevant groupings	N/A
Population characteristics	N/A
Recruitment	N/A
Ethics oversight	N/A

Note that full information on the approval of the study protocol must also be provided in the manuscript.

Field-specific reporting

Please select the one below that is the best fit for your research. If you are not sure, read the appropriate sections before making your selection.

[x] Life sciences [ ] Behavioural & social sciences [ ] Ecological, evolutionary & environmental sciences

For a reference copy of the document with all sections, see nature.com/documents/nr-reporting-summary-flat.pdf

Life sciences study design

All studies must disclose on these points even when the disclosure is negative.

Sample size	For optical density measurements of bacterial growth or lysis, the sample size was the number of parallel cell cultures. Under the null hypothesis that the ΔbamD mutant and its suppressors have the same growth/lysis phenotype as the parent strain, we determined that 3 biological replicates were sufficient to reject the null hypothesis when there was a large change in growth/lysis, based on a two-sided Student's t-test with statistical significance of 0.05 and a power of 0.90.<br><br>For the measurement of NPN fluorescence for OM permeability, the sample size is the number of parallel cell cultures. Under the null hypothesis that the ΔbamD mutant and its suppressors have the same NPN intake, we determined that 3 biological replicates were sufficient to reject the null hypothesis when there was a large change in the fluorescence intensity, based on a two-sided Student's t-test with statistical significance of 0.05 and a power of 0.90.<br><br>For growth rate analyses, the sample size was the number of cells analyzed. Under the null hypothesis that the ΔbamD mutant has the same single cell growth rate as the parent strain, we determined that 100 cells were sufficient to reject the null hypothesis when there is a change in growth rate of around 10%, based on a two-sided Student's t-test with statistical significance of 0.05 and a power of 0.90. The actual sample sizes of each cell width group were determined based on the calculation and the actual number of cells we could measure in one experiment.<br><br>For cell length measurements to determine OM stiffness, the sample size is the number of cells analyzed. Under the null hypothesis that the ΔbamD mutant and its suppressors have the same OM stiffness as the parent strain, we determined that 10 cells were sufficient to reject the hypothesis when there was a 50% change in the length contraction, based on a two-sided Student's t-test with statistical significance of 0.05 and a power of 0.90.
Data exclusions	No data were excluded from analysis.
Replication	All measurements were reliably reproduced.
Randomization	No randomization was carried out, as this was not relevant to the standard bacterial culturing and imaging methods applied in our work. Covariates were thus not relevant to our work.
Blinding	No blinding was carried out, as this was not possible due to the need to culture each bacterial strain with specific protocols.

Reporting for specific materials, systems and methods

We require information from authors about some types of materials, experimental systems and methods used in many studies. Here, indicate whether each material, system or method listed is relevant to your study. If you are not sure if a list item applies to your research, read the appropriate section before selecting a response.

Materials & experimental systems

n/a	Involved in the study
[ ]	[x] Antibodies
[x]	[ ] Eukaryotic cell lines
[x]	[ ] Palaeontology and archaeology
[x]	[ ] Animals and other organisms
[x]	[ ] Clinical data
[x]	[ ] Dual use research of concern
[x]	[ ] Plants

Methods

n/a	Involved in the study
[x]	[ ] ChIP-seq
[x]	[ ] Flow cytometry
[x]	[ ] MRI-based neuroimaging

Antibodies

Antibodies used	Polyclonal antisera against outer membrane proteins were generated for this work by the Thomas Silhavy lab at Princeton. Monoclonal antibodies against GroEL (Cat. #G6532) and FLAG (Cat. #F3165) were products from Sigma-Aldrich. Goat anti-rabbit IgG horseradish peroxidase (Cat. #12-348) and goat anti-mouse IgG horseradish peroxidase (#1706516) were also products from Sigma-Aldrich.
Validation	Validation of antisera against outer membrane proteins is based on previous work by the Silhavy lab.
